# The effect of Korean pine nut oil on in vitro CCK release, on appetite sensations and on gut hormones in post-menopausal overweight women

**DOI:** 10.1186/1476-511X-7-10

**Published:** 2008-03-20

**Authors:** Wilrike J Pasman, Jos Heimerikx, Carina M Rubingh, Robin van den Berg, Marianne O'Shea, Luisa Gambelli, Henk FJ Hendriks, Alexandra WC Einerhand, Corey Scott, Hiskias G Keizer, Louise I Mennen

**Affiliations:** 1 Businessunit Biosciences, TNO Quality of Life, PO box 360, 3700 AJ Zeist, The Netherlands; 2 Lipid Nutrition B.V., PO box 4, 1520 AA Wormerveer, The Netherlands; 3 Businessunit Quality and Safety, TNO Quality of Life, PO box 360, 3700 AJ Zeist, The Netherlands; 4 Mennen Training & Consultancy, Junoplantsoen 127, 2024 RP Haarlem, The Netherlands; 5 LM currently works for Mennen training & Consultancy. RvdB and AWCE changed jobs after having contributed to this paper

## Abstract

Appetite suppressants may be one strategy in the fight against obesity. This study evaluated whether Korean pine nut free fatty acids (FFA) and triglycerides (TG) work as an appetite suppressant. Korean pine nut FFA were evaluated in STC-1 cell culture for their ability to increase cholecystokinin (CCK-8) secretion vs. several other dietary fatty acids from Italian stone pine nut fatty acids, oleic acid, linoleic acid, alpha-linolenic acid, and capric acid used as a control. At 50 μM concentration, Korean pine nut FFA produced the greatest amount of CCK-8 release (493 pg/ml) relative to the other fatty acids and control (46 pg/ml). A randomized, placebo-controlled, double-blind cross-over trial including 18 overweight post-menopausal women was performed. Subjects received capsules with 3 g Korean pine (*Pinus koraiensis*) nut FFA, 3 g pine nut TG or 3 g placebo (olive oil) in combination with a light breakfast. At 0, 30, 60, 90, 120, 180 and 240 minutes the gut hormones cholecystokinin (CCK-8), glucagon like peptide-1 (GLP-1), peptide YY (PYY) and ghrelin, and appetite sensations were measured. A wash-out period of one week separated each intervention day.

CCK-8 was higher 30 min after pine nut FFA and 60 min after pine nut TG when compared to placebo (p < 0.01). GLP-1 was higher 60 min after pine nut FFA compared to placebo (p < 0.01). Over a period of 4 hours the total amount of plasma CCK-8 was 60% higher after pine nut FFA and 22% higher after pine nut TG than after placebo (p < 0.01). For GLP-1 this difference was 25% after pine nut FFA (P < 0.05). Ghrelin and PYY levels were not different between groups. The appetite sensation "prospective food intake" was 36% lower after pine nut FFA relative to placebo (P < 0.05).

This study suggests that Korean pine nut may work as an appetite suppressant through an increasing effect on satiety hormones and a reduced prospective food intake.

## Background

The global epidemic rise in obesity urgently requests new strategies that are effective and easy to apply for the general population. One potential strategy in the fight against obesity is to reduce food intake via the use of natural appetite suppressants. It has been shown that by overeating by as little as 50 to 100 kcal above energy balance it is possible to gain at least one kilo a year [1] Thus, preventing the surplus in energy intake through natural appetite suppressants may be useful in the prevention of obesity.

Food intake induces a complex feed back system regulating hunger and satiety. Intake, digestion and absorption of food are all regulated by the nervous and the hormonal system [2]. Several important gastro-intestinal hormones exist of which some are known to be induced by fat intake. A well-studied gut hormone is cholecystokinin (CCK-8) that is released from duodenal enteroendocrine cells into the blood stream in response to fatty acids or protein [3]. CCK-8 suppresses appetite, and higher concentrations of CCK-8 produce larger appetite-suppressing events [4,5]. Other hormones that are important are glucagon like peptide-1 (GLP-1), peptide YY (PYY) and ghrelin [6-9]. GLP-1 and PYY are both produced in the ileum, induced by passing fatty acids and carbohydrates. Higher concentrations decrease appetite [6,9]. Ghrelin is produced by the stomach in reaction to macronutrients [8]. It is the only 'satiety' hormone, which induces hunger, i.e. higher concentrations of ghrelin increase appetite. It is therefore called the "hunger" hormone.

The satiety hormones increase after fat intake. Fat digestion leads to formation of mono-glycerides and fatty acids. Only fatty acids with chain lengths ≥ C12 are capable of releasing CCK-8 [10,11]. Long chain fatty acids are more effective than medium chain fatty acids and poly-unsaturated fatty acids are more effective than mono-unsaturated fatty acids [12,13].

The purpose of this study was to evaluate the effects of long chain poly unsaturated fatty acids from Korean pine (*Pinus koraiensis*) nuts on satiety. Nut consumption has been previously linked to satiety and pine nuts are widely consumed in popular dishes and as condiments [14-17]

Oils are major constituents of nuts with the oil consisting of as much as 60% of weight in pine nuts. Korean Pine nut oil consists of more than 92% of poly- and mono-unsaturated fatty acids (PUFAs and MUFAs) like pinolenic acid (C18:3), linoleic acid (C18:2) and oleic acid (C18:1) [14]. Korean Pine nut oil is unique in that it contains approximately 15% of pinolenic acid (C18:3). Previous studies on Korean pine nut oil have shown beneficial effects such on lipoprotein metabolism and immune function but the effects on satiety hormones have not been studied. [18]. The high composition of poly unsaturated fats and unique concentration of pinolenic acid leads to the hypothesis that Korean pine nut oil may be able reduce appetite by an induction of satiety hormones. The present study was designed to evaluate the effects of Korean pine nut oil on in vitro CCK-8 release and if Korean Pine nut oil is also capable of releasing satiety hormones in humans and thereby having an effect on appetite sensations.

## Results

### Effects of Fatty acids on in vitro CCK release

**Figure 1:**
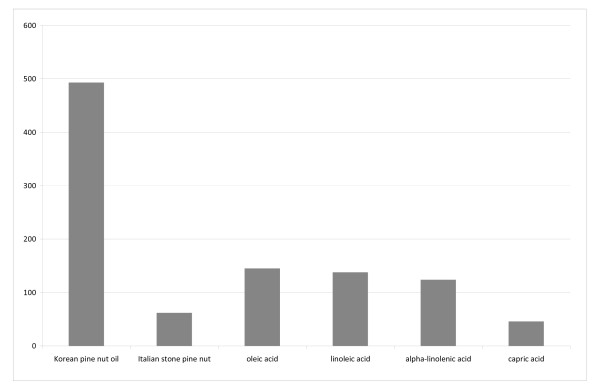
**Free fatty acids of Korean pine nut oil (Pinnothin), Italian stone pine nut and the fatty acids oleic acid, linoleic acid, alfa linolenic acid were compared with respect to CCK release (ng/ml) from STC-1 cells after exposure for 1 hour to 50 uM of the fatty acids. **Capric acid served as a negative control.

The fatty acids evaluated in this study produced different levels of CCK-8 release in the STC-1 cells (Figure 1). Carpic acid produce a very small amount of CCK-8 release (46 pg/ml) relative the the other fatty acids whereas Korean pine nut fatty acids produced the largest amount of CCK-8 release (493 pg/ml). The other fatty acids oleic acid, linoleic acid, alpha-linolenic acid, and Italian stone pine nut fatty acids produced 145 pg/ml, 138 pg/ml, 124 pg/ml, and 62 pg/ml respectively.

### Effects of Korean pine nut oil on satiety hormones and subjective measures of satiety

The baseline characteristics of the 18 women that completed the study are presented in Table 1.

**Table 1:** Baseline characteristics of the subjects (n = 18)

**Females**	**Mean ± SEM**
Age (years)	55 ± 1
Body weight (kg)	76.7 ± 1.6
Height (m)	1.68 ± 0.01
BMI^a ^(kg/m^2^)	27.1 ± 0.4
Glucose (mmol/L)	5.3 ± 0.1
Insulin (mU/L)	5.8 ± 0.7
FSH^b ^(IU/L)	101.9 ± 6.1
Oestradiol (pg/mL)	10.0 ± 0.0

^a^BMI = Body Mass Index

^b^FSH = Follicle Stimulating Hormone

**Figure 2:**
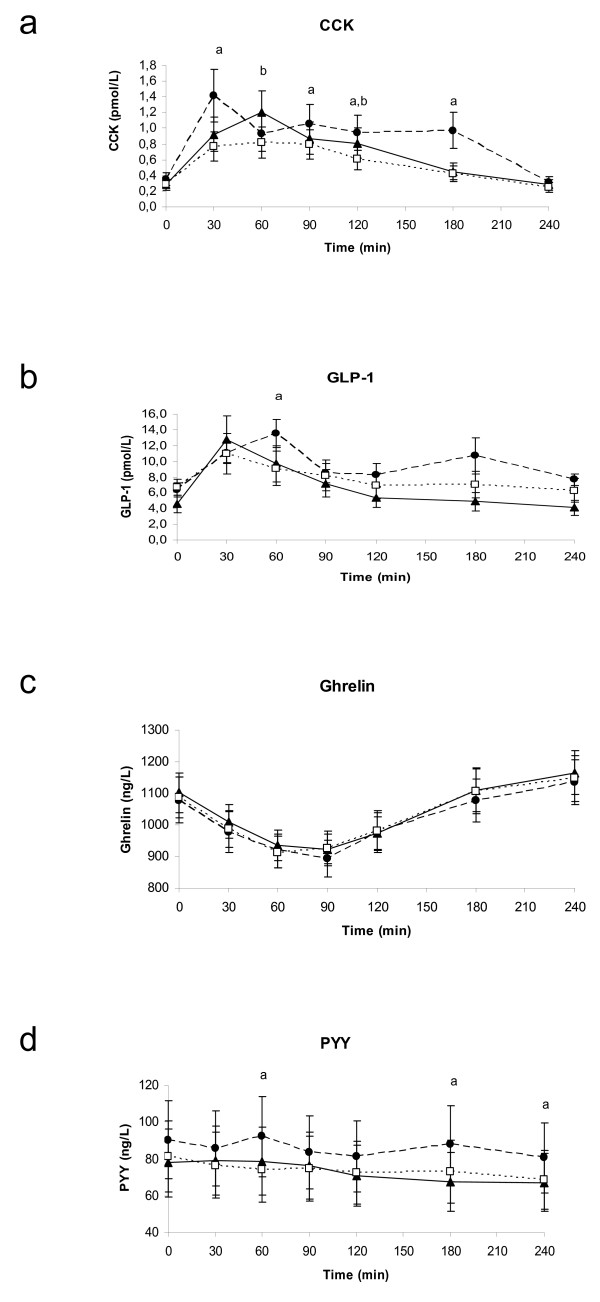
**Four hour postprandial responses of CCK-8 (1a), GLP-1 (1b), ghrelin (1c) and PYY (1d) in 18 overweight women.** (closed circles) Pine nut FFA, (closed triangles) pine nut TG, (open squares) placebo. Significant differences (p < 0.05) indicated by a: pine nut FFA versus placebo, b: pine nut TG versus placebo.

Log-transformation of the data was applied on CCK-8, GLP-1, PYY and TG. CCK-8 release was higher after pine nut FFA than after placebo at 30, 90, 120, and 180 minutes (Figure 2a; p-value: 0.0066; 0.0031; 0.0003; 0.0117; respectively). CCK-8 release was also higher at 60 and 120 minutes for pine nut TG compared with placebo (p-value: 0.0068 and 0.0148). The CCK-8 AUC for pine nut FFA was 60.3% higher than for placebo (p < 0.0001) and for pine nut TG 22.0% higher than the CCK-8 AUC for placebo (p: 0.0189) (Table 2). Different response curves were present for the three different treatments (p < 0.01). The maximal concentration (= Cmax) of CCK-8 reached was for pine nut FFA 2.0 ± 0.43 pmol/L, for pine nut TG 1.45 ± 0.23 pmol/L and for placebo 1.08 ± 0.15 pmol/L (p-value FFA vs placebo: 0.0001; p-value TG vs placebo: 0.0358; analysis based on log transformed data).

**Table 2:** Total area under the curve of the satiety hormones (n = 18)

**Hormone**	**Treatment**	**AUC total**^**a**^	**p-value**^**b**^
CCK-8	Pine nut FFA	217.4 ± 28.1	< 0.0001^c^
	Pine nut TG	165.4 ± 20.2	
	Placebo	135.6 ± 14.0	
GLP-1	Pine nut FFA	2340.5 ± 268.3	0.0037^c^
	Pine nut TG	1624.2 ± 130.3	
	Placebo	1871.3 ± 174.9	
Ghrelin	Pine nut FFA	241892 ± 14916	0.3966
	Pine nut TG	248000 ± 13517	
	Placebo	246133 ± 14685	
PYY	Pine nut FFA	20581 ± 1419	0.0060^c^
	Pine nut TG	17485 ± 1100	
	Placebo	17707 ± 918	

^a^All values are means ± SEM.

^b^Differences between the three treatments were tested with a one-way ANOVA.

^c^Analysis based on log-transformed data.

GLP-1 (Figure 2b) also showed overall different response curves (p < 0.01) between treatments. The GLP-1 release was higher after pine nut FFA then after the placebo at 60 (p-value: 0.0101). GLP-1 AUC was 25.1% higher after the pine nut FFA than after placebo (p-value: 0.0343).

For ghrelin similar curves were seen after all three treatments (Figure 2c) and no effect of pine nut FFA or pine nut TG were observed.

At 60, 180 and 240 minutes the PYY concentrations after pine nut FFA was higher than after placebo (Figure 2d). The AUC was 16.2% higher for pine nut FFA versus placebo (p < 0.01).

**Figure 3:**
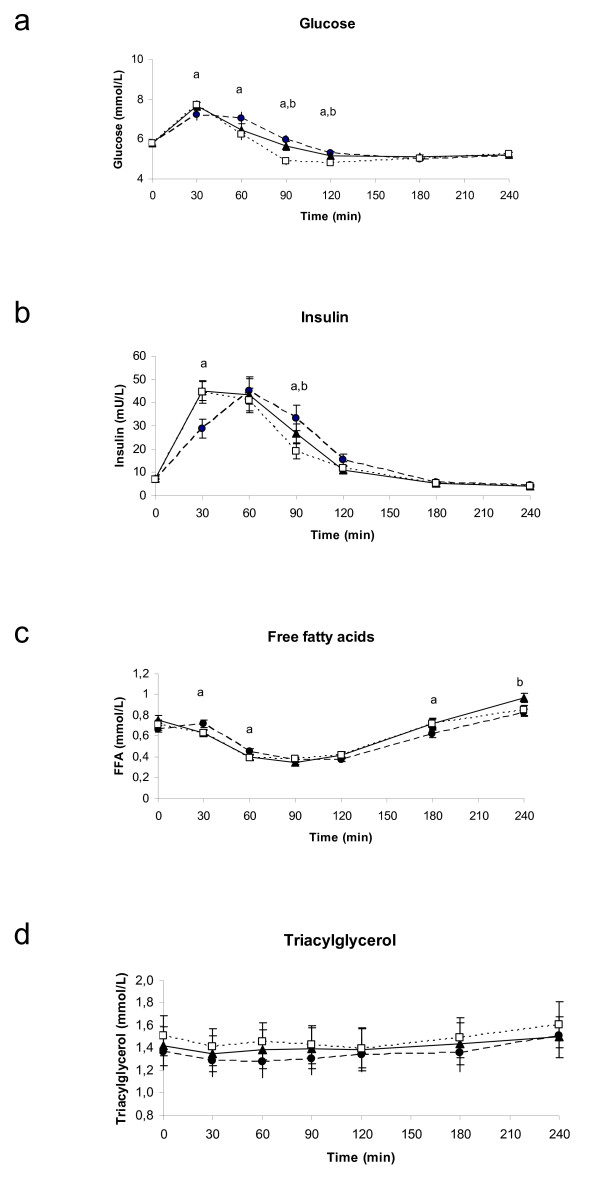
**Four hour postprandial responses of glucose (2a), insulin (2b), serum free fatty acids (2c) and serum triglycerides (2d) in 18 overweight women.** (closed circles) Pine nut FFA, (closed triangles) pine nut TG, (open squares) placebo. Significant differences (p < 0.05) indicated by a: pine nut FFA versus placebo, b: pine nut TG versus placebo.

The curves of blood glucose, insulin, serum TG and FFA showed normal physiological responses to a carbohydrate-rich breakfast with no or only marginal differences between the treatments (Figure 3a–3d). For glucose the response differed slightly between pine nut FFA and placebo at 30, 60, 90 and 120 minutes (Figure 3a; p-values: 0.0077; 0.0021; <0.0001; 0.0044; respectively). At 90 and 120 minutes also the response after pine nut TG was slightly different from placebo (p-value: 0.0004 and 0.0230). This difference was reflected in a slightly higher AUC after pine nut FFA compared to placebo (Table 3). The insulin response was lower after 30 and higher at 90 minutes after pine nut FFA compared to placebo (Figure 3b) (p < 0.05), but no differences were seen in the AUC. The insulin response at 90 minutes was also higher for pine nut TG compared to placebo (p < 0.05). The serum FFA concentrations showed only minor differences at three time points for pine nut FFA (at 30, 60 and 180 minutes; p-value: 0.0027; 0.0200; 0.0437; respectively) and for the last time point for pine nut TG versus placebo (p-value = 0.0210; Figure 3c), but no difference in AUC. There were no differences between the treatments for serum TG (Figure 3d).

**Table 3:** Total area under the curve of blood parameters (n = 18)

**Parameter**	**Treatment**	**AUC total**^**a**^	**p-value**^**b**^
Glucose	Pine nut FFA	1386.9 ± 29.8	0.0012
	Pine nut TG	1376.4 ± 27.3	
	Placebo	1329.8 ± 24.4	
Insulin	Pine nut FFA	4482.8 ± 414.7	0.3393
	Pine nut TG	4492.7 ± 420.4	
	Placebo	4210.2 ± 369.7	
Free fatty acids	Pine nut FFA	135.1 ± 4.7	0.1849
	Pine nut TG	144.3 ± 6.4	
	Placebo	139.8 ± 6.0	
Triglycerides	Pine nut FFA	324.0 ± 37.1	0.4787^c^
	Pine nut TG	339.5 ± 43.2	
	Placebo	352.0 ± 41.8	

^a^All values are means ± SEM.

^b^Overall differences between the three treatments were tested with a one-way ANOVA.

^c^Analysis based on log-transformed data.

**Figure 4:**
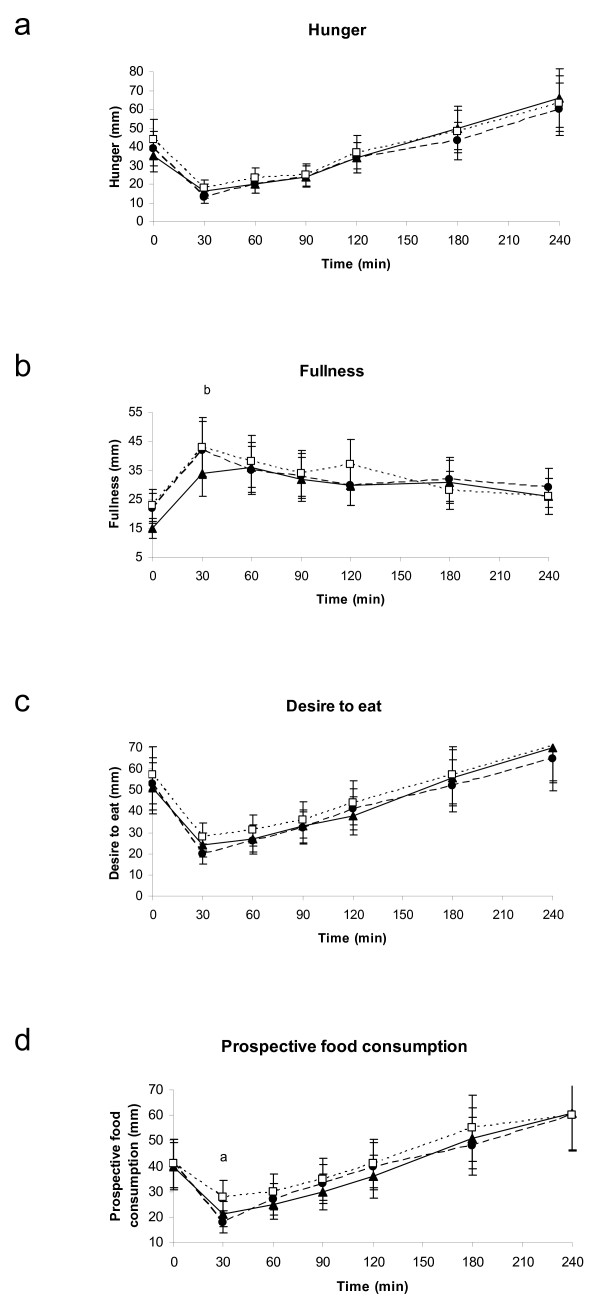
**Mean four hour responses of hunger (3a), fullness (3b), desire to eat (3c) and prospective food consumption (3d) in 18 overweight women.** (closed circles) Pine nut FFA, (closed triangles) pine nut TG, (open squares) placebo. Significant differences (p < 0.05) indicated by a: pine nut FFA versus placebo, b: pine nut TG versus placebo.

With respect to the appetite scores, no differences were present between the treatments for hunger or desire to eat (Figures 4a and 4c). Fullness was lower after pine nut TG than after placebo at 30 minutes (p < 0.01), but this was not the case for pine nut FFA (Figure 4b). The overall AUC was however similar for the three treatments (Table 4). Prospective food consumption was at 30 minutes 36% lower for pine nut FFA compared to placebo (p-value: 0.0068) and, also pine nut TG seemed to be lower (p-value = 0.0559; Figure 4d).

**Table 4:** Total area under the curve of the VAS scores (n = 18)

**VAS score**	**Treatment**	**AUC total**^**a**^	**p-value**^**b**^
Hunger	Pine nut FFA	8211 ± 614	0.4697
	Pine nut TG	8819 ± 886	
	Placebo	9100 ± 892	
Fullness	Pine nut FFA	7770 ± 832	0.5326
	Pine nut TG	7273 ± 681	
	Placebo	7915 ± 633	
Desire to eat	Pine nut FFA	10039 ± 719	0.2241
	Pine nut TG	10477 ± 896	
	Placebo	11252 ± 790	
Prospective food consumption	Pine nut FFA	9438 ± 703	0.2763
	Pine nut TG	9372 ± 754	
	Placebo	10323 ± 687	

^a^All values are means ± SEM.

^b^Overall differences between the three treatments were tested with a one-way ANOVA.

No adverse events were reported that were caused by the treatments.

## Discussion

This study shows for the first time that Korean pine nut FFA and TG can increase satiety hormones and thereby may affect prospective food intake.

Nut consumption is popular worldwide and has shown many health benefits, particularly against cardiovascular disease. [19,20] It has also been shown that nut consumption is related to satiety and epidemiological evidence links nut consumption inversely with body weight. [20] Knowing that nuts can have effects on satiety, the purpose of this trial was to evaluate the effects of oil from Korean pine nuts and several dietary oils on *in vitro *and *in vivo *satiety hormone release and feelings of satiety. It was observed from the STC-1 cell culture study the fatty acids from Korean pine nuts had the greatest ability to increase CCK-8 release compared to the other fatty acids tested and capric acid, the negative control. Korean pine nut FFA were approximately 8-fold more potent in releasing CCK-8 than Italian stone pine nut FFA, although they have similar fatty acid profiles. However, Korean pine nut oil does contain slightly more long chain poly unsaturated fatty acids (which contribute to CCK release) and is much higher concentrated with pinolenic acid (15% vs. 1%). The large discrepancy in pinolenic acid concentration may be responsible for the large difference in CCK release between Italian stone pine nut FFA and the other FFA tested. Oleic, linoleic, and alpha-linolenic acid produced similar amounts of CCK-8 release which were all approximately 2-fold greater than Italian stone pine nut FFA. A potential reason for this difference may be the specific fatty acid concentration in the dosage, as the Italian stone pine nut FFA was a 50 μM dose of a mixture of fatty acids whereas the others was a single 50 μM dose of each FFA.

The fact that Korean pine nut FFA had the ability to significantly increase satiety hormones in vitro lead to the analysis in human subjects. The in vivo arm of the study was performed in postmenopausal overweight women. The results can not easily be applied to the total population (men and women; lean and obese; pre-and postmenopausal women) and therefore needs to be confirmed in other studies as well. The subjects in this study were all 100% compliant as the capsules were always taken in the presence of the study nurse. As there were no differences in dinner consumption at home the evening prior to the test day (data not shown) we assume that this did not affect the hunger and satiety scores in the morning.

The effect of pine nut FFA on CCK-8 was stronger than for pine nut TG. This is consistent with literature stating that FFA are needed for induction of CCK-8 [21-23] From each TG only two FFA will be hydrolyzed during digestion in the gut. Therefore supplementation with pure FFA will lead to a higher amount of FFA in the gut than pure TG and thus to a higher effect on CCK-8. We could speculate that 1.5 times more pine nut TG is required relative to pine nut FFA to induce a similar increase in satiety hormones.

GLP-1, like CCK-8, is known to be a potent regulator of food intake; peripheral administration of GLP-1 reduces energy intake in a dose dependent manner in both lean and overweight human subjects [9]. Both CCK-8 and GLP-1 release lead to a delay in gastric emptying, early satiety and a decrease in food consumption [24]. They serve as a negative feedback signal to limit the amount of food consumed [25,26]. The effect of pine nut FFA and TG on these hormones seen in this study suggests therefore that they may influence appetite. As far as we know, no other study has been performed so far to confirm these results.

PYY release is known to be stimulated by lipids depending on the fatty acid chain length (≥ C12) [6,27]. However, in this study we could not demonstrate an effect of pine nut FFA or TG on PYY, probably due to basal increased PYY levels with pine nut FFA, although not statistically significant. The shape of the PYY curve after pine nut FFA was similar to the CCK-8 curve. This might be due to the effect of these hormones on gastric emptying and gut transit time slowing down the gastrointestinal passage of nutrients [28,29]. It has been reported that at least in dogs CCK-8 induction coincided with PYY release after fat intake [30].

There was no difference in effect on ghrelin between pine nut FFA or TG and placebo. This is in line with a recent study by Poppitt and coworkers [31] showing that the high fat meals and fatty acid saturation levels had no differential effect on ghrelin levels in healthy men.

The overall glucose levels were slightly higher within 4 hours after pine nut FFA than after placebo. However, the glucose levels stayed within normal ranges and returned to levels identical to the placebo group within 3 hours. In addition, only minor changes were seen in postprandial insulin and serum FFA concentrations after the treatments, but were not consistent over time.

Overall appetite sensations after the breakfast meal were only marginally affected. Due to the smaller effects present than expected and the high variability in VAS, as is often seen in the subjective VAS scores [32,33], it seems likely that this study was underpowered to demonstrate a clear and significant effect on VAS scores.

The mechanism through which pine nut FFA and TG are able to induce CCK-8 and GLP-1 remains unknown. The formation or transport of chylomicrons might play a role. It is known that fatty acids with chain lengths of <12 are transported from the enterocyte directly into the systemic circulation via the portal vein, whereas fatty acids with chain lengths of ≥ 12 are absorbed into the lymphatic circulation as chylomicrons [34,35]. When chylomicron transport was blocked in rats by specific inhibitors, fat-induced suppression of food intake was also blocked [36,37]. CCK-8 signaling pathways are closely related to the transport of chylomicrons [38]. The fact that only fatty acids with at least or more than 12 carbon atoms induce CCK-8 is in favor of this mechanism. This suggests that pine nut FFA and TG may particularly affect chylomicron formation or transport and thereby influence release of CCK-8. This fast CCK-8 release activates the CCK_A _receptors of the afferent fibers of the nervus vagus sending satiety signals to the brain which reduces gastric emptying. This limits further food intake through an increased feeling of satiety and a decreased appetite [21,24].

## Conclusion

Pine nut FFA and pine nut TG supplementation seem to increase the satiety-inducing hormones CCK and GLP-1 concentrations postprandially. This may lead to a reduced prospective food intake suggesting that pine nut FFA and TG may act as an appetite suppressant in overweight women. Further human studies are needed to confirm these effects and to evaluate effects on actual food intake and body weight.

## Materials and methods

### Cholecystokinin Release Cell Assay

STC-1 cells were used in this study to evaluate the ability of Korean Pine nut fatty acids and other dietary fatty acids on CCK-8 satiety hormone release. Characterization of the release of cholecystokinin was performed in a murine neuroendocrine tumor cell line, STC-1 [39,40]. All fatty acids were obtained from Lipid Nutrition, B.V. (Wormerveer, The Netherlands). Fatty acids tested are common to the diet and were Korean Pine Nut fatty acids (monounsaturated-24%, polyunsaturated-64%, including pinolenic acid-15%, saturated and trans-12%), Italian Stone Pine nut fatty acids (monounsaturated-32%, polyunsaturated-59% including 1% pinolenic acid, saturated and trans-9%) oleic acid, linoleic acid, alpha-linoleic acid, and capric acid (C-10 fatty acid) negative control. Fatty acids were diluted in cell culture media to a concentration of 50 μM and added to cell culture. STC-1 cells were grown in DMEM containing 2.5% fetal bovine serum, 15% horse serum, 100 U/ml penicillin, and 100 μg/ml streptomycin. Cells were maintained at 37°C in 5% CO_2 _and 95% air atmosphere in RPMI 1640 supplemented with 5% FCS, 2 mM glutamine, 100 U/ml penicillin and 50 μM streptomycin. Media was changed every 3–4 days. Cells were routinely passaged upon reaching 70–80% confluency by washing the cell layer with PBS and incubating with a solution of trypsin-EDTA. Plating density of 2 × 10^6 ^cells by 75 cm^2 ^is used for routine subculture. Cells were trypsinized, plated in six-well culture plates, and allowed to reach 60–80% confluence. On the day of the experiment, cells were washed twice with media (without serum) and then incubated with fatty acids in media for 60 minutes. After a 60 minute incubation period, media were harvested, clarified by centrifugation to remove cell debris and assayed for CCK concentrations using a CCK immunoassay. All fatty acids were tested in at least two separate experiments. 24 h before experiments, STC-1 cells were seeded into 6-well culture plates at 40 – 50% confluency. On the day of the experiment, cells were washed in 1 ml Krebs – Ringer bicarbonate buffer (pH 7,4) containing 0,2% (wt/vol) BSA (KRBB). STC1 cells were pre- incubated for 15 min in 2 ml KRBB, before to be incubated in a 2-ml amount of KRBB, with or without the tested agents for 1 hour.

At the end of incubation the supernatant was collected and centrifuged at 1000 rpm for 5 min and immediately frozen at -20°C for RIA

DNA content was measured by fluorometry after extraction of the culture cell contents.

### CCK immunoassay

Media levels of CCK were measured using a standard immunoassay protocol (Regul Pept 8:97–103) and an antiserum that detects CCK. Media specimens were assayed in duplicate. CCK immunoreactivity was measured using antiserum .39A (1/300,000) that cross reacts 100% with CCK-33 and CCK-8, 12% with sulfated gastrin-17, 5% with unsulfated gastrin-17, and less than 0.1% with unsulfated CCK-8 and gastrin-34. CCK-8 was used as standard.

The Bolton-Hunter (Thr, Nle)-CCK-9 was labelled with iodine 125 by the chloramine-T method and purified by reverse-phase HPLC. The assay buffer used was 0.05 M sodium phosphate pH 7.5.

Aliquots of 1 μl to 200 μl of supernatant were tested in duplicate (day 0). The label was added at day 1 and charcoal precipitation was performed 48 h later.

### Subjects for in vivo CCK release

In total, 48 candidates were invited from the pool of volunteers of TNO Quality of Life and came to the study information session. After signing the informed consent forms, 31 subjects received a health screening. This included an interview on medical history, physical examination and routine laboratory tests on blood and urine, sampled after an overnight fast. Subjects needed to meet the following inclusion criteria: at least two years post-menopausal with follicle stimulating hormone (FSH) > 40 IU/L and Oestradiol < 70 pg/mL; body mass index (BMI) between 25–30 kg/m^-2^; regular eating pattern; no metabolic, endocrine, intestinal disorder or a medical history related to the study outcome parameters; no smoking; not sporting > 5 hours/week. Subjects were postmenopausal to exclude a possible influence of the menstrual cycle on appetite. Twenty-five subjects were eligible for the study. Based on power calculations on appetite scores it was found that for a power of 80%, 18 subjects should be included. Therefore, 18 subjects were assigned to start the study and three were appointed as reserve. At the first day of the study one subject dropped out, because cannula insertion failed. She was immediately replaced by a reserve subject.

The study was approved by the Medical Ethics Committee of the University Medical Centre of Utrecht (February 22, 2005) and conducted according to the ICH Guideline for Good Clinical Practice, in March 2005.

### Study design

The study was a randomised, placebo-controlled, double blind cross-over trial. The cross-over design was balanced for carry over. The participants were randomly assigned to the intervention groups, with age and BMI equally distributed over the treatment groups.

### Study treatment

Treatment consisted of either 3 g Korean pine nut FFA, 3 g Korean pine nut TG or 3 g olive oil (placebo) which was delivered by Lipid Nutrition B.V. (PinnoThin™, The Netherlands). The treatments were delivered in the form of soft gel capsules, with one capsule containing 750 mg. Subjects were therefore provided each treatment with four capsules to consume the total dose of 3 gram. The fatty acid composition (%) of pine nut FFA, pine nut TG and olive oil respectively was as follows: pinolenic acid 15.2%, 15.3%, 0.0%; linolenic acid 0.0%, 0.0%, 0.7%; linoleic acid 45,1%, 45.6%, 5.6%; oleic acid 26.8%, 26.4%, 77.4 and saturated fatty acids 7.2%, 6.9%, 11.7%.

### Study procedures

Subjects came to the metabolic ward of TNO Quality of Life, Zeist, after an overnight fast. A cannula was inserted in the forearm in an antecubital vein and a blood sample was obtained (t = 0). The subjects were then provided with a breakfast together with four treatment capsules. The breakfast contained two slices of white bread with marmalade and without margarine. No additional fat besides the capsules was provided. The subjects consumed the breakfast and capsules together with one glass of water within 10 minutes. The composition of the breakfast was: energy content: 954 kJ (228 kcal); protein: 5 grams (8.6 in energy percentage (en%); fat: 1 gram (5.1 en%); carbohydrates: 49 grams (86.3 en%); fiber: 1.9 grams. After capsules and breakfast were taken, blood was sampled at t = 30, 60, 90, 120, 180 and 240 minutes after start of breakfast. Appetite feelings were scored each time just after blood collection was performed. These procedures were followed for each treatment on three test days, with a wash-out period of one week between treatments.

### Blood parameters

For blood lipids, glucose and insulin, blood was collected using Vacutainer^® ^tubes (10 mL). To obtain serum, the tubes contained clot activator and were centrifuged for 10 minutes within 30 min after blood collection. The centrifuge was adjusted at 2,000 g and 4°C. After centrifugation serum was removed. Samples were stored at -18°C, until analysis. Glucose, TG and FFA were determined using commercial test kits (Boehringer, Mannheim, Germany) on a Hitachi 911 automatic analyser (Hitachi Instrument Division, Ibaraki-ken, Japan). Insulin was determined using an AIA-600 Immunoassay Analysator.

For satiety hormones blood was collected in a Vacutainer^® ^tube (10 ml) containing K_3_EDTA as coagulant and put on ice water. Protease inhibitor Aprotinin (at a final concentration of 500 KIU/ml of blood) and a DPP-IV inhibitor (10 μl per ml of blood) (LINCO Research, Missouri, USA) was immediately added to the blood. Subsequently, samples were centrifuged for 15 minutes at 2,000 g at 4°C, within 30 minutes after collection and stored at < -70°C, until analysis. Plasma CCK-8 concentrations were measured using an optimized and validated commercial human RIA kit (Euro-Diagnostica, Malmö, Sweden). This CCK-8 assay system has been optimized to reach a sensitivity of 0.05 pmol/L with no cross-reactivity towards gastrin-17, and sulphated gastrin. The intra-assay CV was 8.9% at a concentration of 0.84 pmol/l and 4.9% at a concentration of 1.98 pmol/L. Plasma total GLP-1 concentrations were measured using an optimized and validated commercial human RIA kit (LINCO Research, Missouri, USA). This assay has been optimized for quantitative determination of all forms of GLP-1 (i.e. GLP-1(7–36) amide, GLP-1(7–37), GLP-1(9–36) amide, GLP-1(9–37), GLP-1(1–36) amide and GLP-1(1–37)) in plasma with a lowest limit of detection of 2.0 pmol/L. The intra-assay CV of GLP-1 was 13.3% at a concentration of 15.6 pmol/L and 4.6% at a concentration of 31.9 pmol/L. Plasma total PYY concentrations were measured using a commercially available human RIA kit (LINCO Research, Missouri, USA). This radioimmunnassay utilizes an antibody, which recognizes both the 1–36 and 3–36 forms of human PYY. The intra-assay CV of PYY was 2.7% at a concentration of 69.7 ng/L and 1.1% at a concentration of 116.4 ng/L.

Plasma total ghrelin concentrations were measured using a commercially available human RIA kit (LINCO Research, Missouri, USA). This total ghrelin assay measures both octanoylated ghrelin and des-octanoylated ghrelin. The intra-assay CV of the total ghrelin RIA kit was 10% at a concentration of 1000 pg/ml, and 3.3% at a concentration of 1500 pg/ml.

### Appetite scores

Appetite was evaluated using visual analogue scales (VAS) for 'hunger', 'fullness', 'desire to eat' and 'prospective food consumption' [32,33]. Visual analogue scales consisted of 150 mm horizontal lines, with each end expressing the most positive or negative sensation (i.e. I am not hungry at all/I am extremely hungry). Subjects drew a vertical line on the horizontal line corresponding to their appetite sensation. Visual analogue scales were scanned using Teleform Elite software (Teleform Elite, Version 6.2, Cardiff Software Inc., California, USA). Distances on the visual analogue scales were converted into scores between 0 and 100.

### Statistical methods

Analysis of variance (one way ANOVA) was used to compare the response curves of CCK-8, GLP-1, PYY, ghrelin, glucose, insulin, serum TG and FFA and the VAS scores after the three treatments. The total area under the curve (AUC) was calculated for all parameters. The curve characteristics, AUC and the maximal concentration (Cmax) were statistically examined. The data were also analysed on treatment effects per time point using one way ANOVA. If the model assumptions were violated (non-normality of the residuals), a log-transformation was applied before the statistical analysis.

All data are presented as mean ± SEM. Statistical analysis of the data was carried out using the SAS statistical software package (SAS/STAT Version 8.2, SAS Institute, Cary, NC). A p-value < 0.05 (two-sided) was considered statistically significant in all analyses.

## Competing interests

Lipid Nutrition is involved in research/development and marketing/sales of Pinnothin as a satiety ingredient. The general goal of Lipid Nutrition is to develop and sell lipids and oils with a scientifically proven health benefit. Pinnothin consists of Korean pine nut oil. Therefore Lipid Nutrition has a commercial interest in this publication. TNO, the conducting laboratory, was paid by Lipid Nutrition to perform and report the scientific work which formed the basis of this publication. TNO and Lipid Nutrition declare that the data presented in this publication represent a true and faithful representation of the work performed.

## Authors' contributions

WJP (of Businessunit Biosciences, TNO Quality of Life, PO box 360, 3700 AJ Zeist, The Netherlands) participated in the study design, in the analyses and interpretation of the data and writing of the manuscript. She is also corresponding author. JH participated in preparing the test materials and in writing of the manuscript. CMR and RvdB were involved in performing the studies, (statistical) analysis and writing of the manuscript. HFJH had final responsibility over the practical part of the study and in writing the manuscript. MO'S, LG, AWCE, were involved in setting up the study design and writing the manuscipt. LIM and CS were involved in the interpretation of the data and writing the manuscript. HGK was involved in writing the manuscript and for submission of this work for publication.
